# Compromised Myelin and Axonal Molecular Organization Following Adult-Onset Sulfatide Depletion

**DOI:** 10.3390/biomedicines11051431

**Published:** 2023-05-12

**Authors:** Elizabeth Dustin, Edna Suarez-Pozos, Camryn Stotesberry, Shulan Qiu, Juan Pablo Palavicini, Xianlin Han, Jeffrey L. Dupree

**Affiliations:** 1 Research Service, Richmond Veterans Affairs Medical Center, Central Virginia Veterans Affairs Health Care System, Richmond, VA 23249, USA; dustine@vcu.edu (E.D.);; 2 Department of Anatomy and Neurobiology, Virginia Commonwealth University, Richmond VA 23298, USA; 3 Department of Biology, Virginia Commonwealth University, Richmond, VA 23298, USA; 4 Sam and Ann Barshop Institute for Longevity and Aging Studies, University of Texas Health Science Center at San Antonio, San Antonio, TX 78229, USA; 5 Division of Diabetes, Department of Medicine, University of Texas Health Science Center at San Antonio, San Antonio, TX 78229, USA

**Keywords:** sulfatide, lipid rafts, sphingolipid, myelin, multiple sclerosis

## Abstract

3-O-sulfogalactosylceramide, or sulfatide, is a prominent myelin glycosphingolipid reduced in the normal appearing white matter (NAWM) in Multiple Sclerosis (MS), indicating that sulfatide reduction precedes demyelination. Using a mouse model that is constitutively depleted of sulfatide, we previously demonstrated that sulfatide is essential during development for the establishment and maintenance of myelin and axonal integrity and for the stable tethering of certain myelin proteins in the sheath. Here, using an adult-onset depletion model of sulfatide, we employ a combination of ultrastructural, immunohistochemical and biochemical approaches to analyze the consequence of sulfatide depletion from the adult CNS. Our findings show a progressive loss of axonal protein domain organization, which is accompanied by axonal degeneration, with myelin sparing. Similar to our previous work, we also observe differential myelin protein anchoring stabilities that are both sulfatide dependent and independent. Most notably, stable anchoring of neurofascin155, a myelin paranodal protein that binds the axonal paranodal complex of contactin/Caspr1, requires sulfatide. Together, our findings show that adult-onset sulfatide depletion, independent of demyelination, is sufficient to trigger progressive axonal degeneration. Although the pathologic mechanism is unknown, we propose that sulfatide is required for maintaining myelin organization and subsequent myelin–axon interactions and disruptions in these interactions results in compromised axon structure and function.

## 1. Introduction

The canonical function of the myelin sheath is axonal insulation to propagate a rapid signal through saltatory conduction. However, the myelin sheath is not merely a static insulator, but a dynamic membrane that provides axonal support through glia–neuron communication [1,2,3]. The insulation properties of myelin are derived both from specific functional domains of the myelin sheath and its biochemical composition [4,5,6,7]. The myelin sheath is unique in that it is composed of 70% lipids and 30% proteins, whereas most plasma membranes have an equal ratio of lipids to proteins [8]. Of those lipids, glycosphingolipids constitute about 30% of total lipids, with the majority being galactosylceramide and sulfatide [9,10]. These lipids create membrane stability due to the presence of long unsaturated and monounsaturated acyl chains, which allow for tight molecular packing, and a high degree of hydroxylation, which enables hydrogen bond formation, facilitating even tighter lipid clustering. The combination of this tight packing and rigid carbon chain structure allows for efficient conduction of the electrical signal down the axon [11,12,13,14].

**Figure 1:**
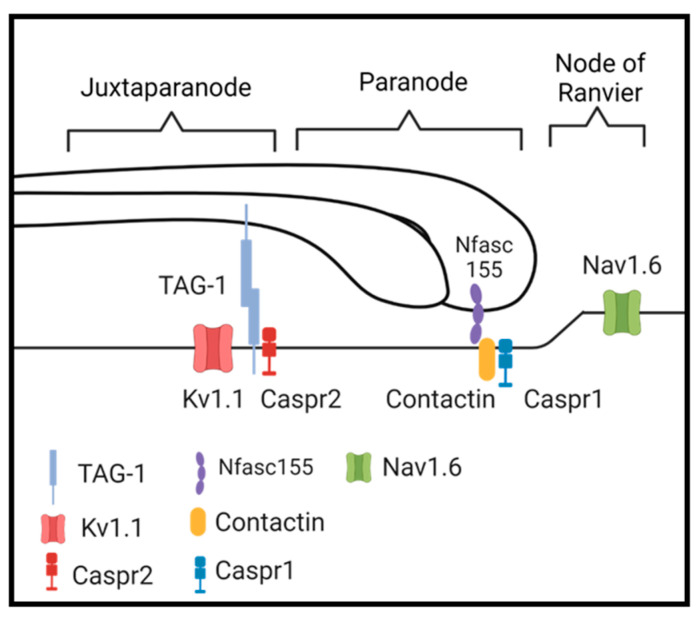
Schematic of relevant proteins localized to their proper domains. Made with Biorender.com.

In addition to its unique composition, the myelin sheath also presents with three distinct domains, each with a specific function. The largest of the domains is known as the internode. The internode presents with tightly compacted concentric wraps of the myelin membranes, yielding the stereotypical structure that is associated with the sheath. Similar to the internode, the juxtaparanode also presents with compacted myelin. The juxtaparanode is positioned at the lateral most extent of the internode and concentrates the adhesion molecule transient axonal glycoprotein-1 (TAG-1) [15,16]. The axonal juxtaparanodal membrane clusters TAG-1 and Caspr2, facilitating homophilic and heterophilic binding with myelin TAG-1 to tether the myelin sheath to the axolemma [15,17,18] (Figure 1). In addition to TAG-1 and Caspr2, voltage-gated potassium channels, which play a role in repolarizing the axon membrane [19,20], also cluster in the axonal juxtaparanode. In contrast to the internode and juxtaparanode which maintain compacted myelin, the sheath also establishes and maintains a domain that is composed of uncompacted myelin known as the paranode. The paranode is structurally demarcated by cytoplasmic sacs, known as the lateral or lateral loops, that align along the axonal membrane [21]. The basal membrane of these loops clusters the myelin specific form of neurofascin, known as neurofascin155 (Nfasc155), while the axonal paranode clusters Caspr1 and contactin, which form a heterodimer that binds the Nfasc155 to form the paranodal junctional complexes that are partially indicated by transverse bands [21,22,23,24] (Figure 1). Positioned between paranodes of adjacent segments of the myelin sheath is the node of Ranvier. Although not a myelin domain, the myelin sheath also maintains additional regions of uncompacted myelin that together are referred to as myelinic channels. These channels include the inner tongue, which is the leading edge of the myelin sheath, the outer tongue, which is the trailing edge of the myelinating membrane and is connected to the oligodendrocyte cell body, and the cytoplasmic channels, which run through the compact myelin [20,25].

The specific mechanisms that regulate the trafficking, sorting and retention of these proteins to their unique destinations of these various domains are not completely defined. Previous work from our group has shown that the myelin sphingolipids, galactosylceramide and its sulfated derivative sulfatide, play a role in these processes [26,27] and in the absence of these lipids, formation and maintenance of protein clusters into myelin domains are compromised [28,29,30]. One plausible explanation for how sphingolipids regulate domain establishment and maintenance involves lipid ordered membrane domains known as lipid rafts (reviewed in [31]). Lipid rafts are enriched in glycosphingolipids and have been implicated in a variety of cellular functions including signal transduction, intercellular adhesion and specific protein sequestering and sorting [32,33,34,35,36,37]. Although lipid rafts may play a role in molecular sorting and ultimately in domain formation and maintenance, it is unclear how certain proteins are included while others are excluded. Our previous studies, using the constitutive knock-out of sulfatide’s synthesizing enzyme, ceramide sulfotransferase (CST), demonstrated that sulfatide, a prominent lipid component of myelin lipid rafts [38], is required for stabilization of certain proteins, namely myelin-associated glycoprotein (MAG) and neurofascin 155 high, within the myelin sheath during development [27]. The identification of these specific proteins is of interest since they have been shown to directly mediate axon–myelin interactions [39,40,41,42]. We hypothesized that these proteins associate with sulfatide in lipid rafts via post translation lipid modifications, including the addition of long saturated fatty chains that form hydrophobic associations with the acyl chain of sulfatide [10].

However, this previous work utilized a constitutive knockout of CST, ablating sulfatide production embryonically [27,43]. To determine if the adult sheath utilizes similar mechanisms to maintain myelin stability, we exploited the LoxP/CreERT system combined with cell-type specific cre expression to deplete sulfatide, specifically in myelinating cells (primarily oligodendrocytes) in adulthood [44,45]. Our findings indicate that adult-onset sulfatide depletion is sufficient to drive axonal pathology and similar to the constitutive CST KO mouse, sulfatide is required for stable tethering of specific myelin proteins within the sheath. Although the tethering stability for most myelin proteins was similar between embryonic and adult sulfatide depletion, some proteins exhibited membrane associations that were apparently dependent on age of extraction.

## 2. Materials and Methods

### 2.1. Animal Model

**Figure 2:**
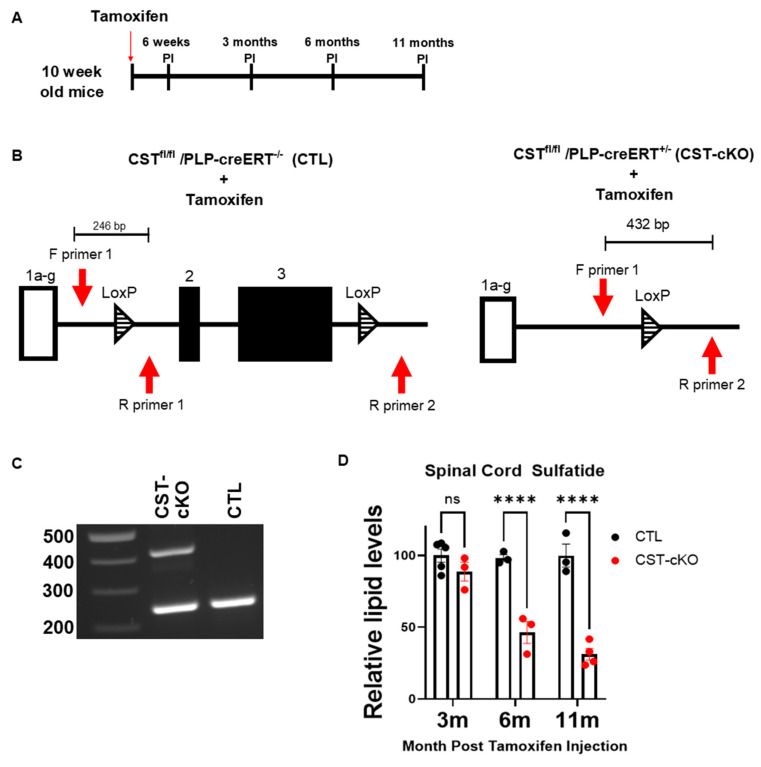
Adult-onset sulfatide depletion model. (**A**) Schematic of the tamoxifen injection paradigm. Both CTL and CST-cKO littermates were aged to 10 weeks and given an intraperitoneal injection of 60 mg/kg of tamoxifen for four consecutive days. Samples were collected at 6 weeks PI for ablation assessment; mice were analyzed at 3, 6 and 11 months PI. (**B**) Schematic illustrating the non-coding (exon 1a-g; white rectangle) and coding (exons 2 and 3; black rectangles) of the *CST* gene with loxP sites (striped triangles) upstream and downstream of coding exons 2 and 3. Three primers (red arrows) were designed around the loxP sites to detect for genetic ablation: F (forward) primer 1, F (forward) primer 2 and R (reverse) primer 1. Tamoxifen was administered to both CST-cKO and CTL mice, resulting in *CST* gene recombination in CST-cKO mice (left) with gene sparing (right) in CTL mice. (**C**) PCR amplification of genomic DNA from the CTL and CST-cKO mice confirmed recombination in CST-cKO mutant mouse brains 6 weeks post tamoxifen injection. (**D**) Lipidomic analysis of total sulfatide in CST-cKO and CTL brains presented no change in sulfatide levels by 3 months post injection, but a significant sulfatide loss at 6 and 11 months post injection. C: **** *p* < 0.0001. All data are presented as mean ± standard error.

To generate a model that enabled adult-onset sulfatide depletion, we contracted with Applied StemCell (Inc, Milpitas, CA, USA) to exploit CRISPR technology to generate a cerebroside sulfotransferase (CST) floxed mouse [44,45]. Upon availability, the CST floxed mouse was crossed with the mouse that expresses Proteolipid Protein (PLP)-creconjugated to a mutated estrogen receptor (ERT) [46] (Jax Labs; stock # 005975) to generate the CST^fl/fl^/PLP-creERT^+/−^ mice (referred to as CST-cKO). The use of the creERT mice provide temporal control of gene ablation thorough the injection of tamoxifen, which induces cretranslocation into the nucleus to promote gene ablation. At 10 weeks old, an age past peak myelination, the CST^fl/fl^/PLP-creERT^+/−^ mice were intraperitoneally injected with 60 mg/kg body weight of tamoxifen (Millipore/Sigma; St. Louis, MO, USA; cat# T5648) for 4 consecutive days. CST^fl/fl^/PLP-creERT^−/−^ mice (referred to as CTL), which were also injected with tamoxifen, constituted the control group for these studies (Figure 2A). Since sulfatide requires 4 months to turn over [47], we analyzed the mice at 3, 6 and 11 months post tamoxifen injection (PI).

### 2.2. Polymerase Chain Reaction

DNA was extracted from the brains of both CST-cKO and CTL mice 6 weeks post tamoxifen injection using Qiagen DNA extraction kit (Qiagen, Germantown, MD, USA, cat# 69504) according to the manufacturer’s instructions. Mice were deeply anesthetized using 0.016 mL/gm body weight of a 2.5% solution of avertin (2, 2, 2 tribromoethanol; Millipore/Sigma; St. Louis, MO, USA; cat#T48402) in 0.9% sodium chloride (Millipore/Sigma, St. Louis, MO; cat# S9888) and transcardially perfused with ice-cold saline. The cervical region of the spinal cord was harvested, snap-frozen in liquid nitrogen and stored at −80 °C until DNA isolation was conducted. DNA fragments spanning genomic target sites were amplified by PCR using the following primers:

 *Gal3st1* Forward (5′-GATTGTAGCCTTCCGTATGAACCG-3′)
 *Gal3st1* Reverse 1 (5′-CGAACTCAACTCAAAGAGAGCAGG-3′) and
 *Gal3st1* Reverse 2 (5′-TAATCTCTGCTCTAACCTGGTCGC-3′).

PCR products were run on a 2% agarose gel at 125 V to electrophoretically separate the products. Gel was visualized on Biorad (Hercules, CA, USA) analyzer imaging software.

### 2.3. Mass Spectrometry

Multidimensional mass spectrometry-based shotgun lipidomics analysis was performed as described [44,45]. Briefly, mice were injected with Avertin and perfused with ice-cold saline. The cervical region of the spinal cord was dissected and flash frozen in liquid nitrogen. Tissue was homogenized in ice-cold phosphate-buffered saline (PBS) 0.1X using Precellys Evolution Tissue Homogenizer (Bertin, France) and protein concentrations was determined using Bio-Rad protein assay (Bio-Rad, Hercules, CA, USA; cat# 5000201). Lipids were extracted following a modified procedure of Bligh and Dyer in the presence of internal standards, which were added based on the total protein content of each sample [44,48]. Lipids were measured using a triple-quadrupole mass spectrometer (TSQ Altis, Thermo Fisher Scientific, Waltham, MA, USA) equipped with a Nanomate device (Advion Ithaca, NY, USA) and Xcalibur system. Sulfatide was assessed by electrospray ionization mass spectrometry (ESI/MS) in the negative-ion mode, ionization voltage was set at −1.15 kV and gas pressure at 0.55 psi as previously described [44,48]. Briefly, nanospray ionization for each sample is performed by a customized sequence subroutine operated under the ChipSoft software (TriVersa NanoMate, Advion Bioscience, Ithaca, NY, USA). Sulfatide species were identified by precursor ion scanning of m/z 98 and directly quantified by comparisons of the individual ion peak intensity with an internal standard, i.e., N16:0 sulfatide (Avanti Polar Lipids, Inc.), which represents <0.1% of the endogenous total sulfatide mass levels in the mammalian CNS. Data were processed using ion peak selection, baseline correction, data transfer, peak intensity comparison, ^13^C deisotoping. Data were analyzed using a custom-programmed Microsoft Excel macro.

### 2.4. Rotarod Assessment

Motor behavior was assessed on the CTL and CST-cKO in mice 8 months PI using the Rotarod (RotaMex-5, Columbus Instruments, Columbus, OH, USA) paradigm and was conducted by the Integrated Physiology of Aging Core of San Antonio Nathan Shock Center. Both male and female mice (*n* = 21 and 23, respectively) were assessed over five consecutive days until they reached maximal performance to eliminate learning effects to complete the task. Eight trials were performed each day. During each trial, the rotarod began at an initial speed of 4 rpm and then increased gradually to 40 rpm within 300 s. Passive Resting (PR) time was also collected as the time the mouse spent still on the rod. Data are plotted as the average of the eight trials per day.

### 2.5. Electron Microscopy

Mice were survived for 3, 6 and 11 months PI and cervical spinal cords were prepared for standard transmission electron microscopic analyses as previously described [30,49]. Briefly, mice were transcardially perfused with 0.1 M Millonigs buffer containing 4% paraformaldehyde and 5% glutaraldehyde and the whole body was post fixed for 2 weeks. Cervical spinal cords (1 mm specimens) were post fixed in 1% osmium tetroxide (Electron Microscopy Sciences, Fort Washington, PA, USA; cat# 19150), dehydrated in ethanol and embedded in PolyBed resin (PolySciences, Warrington, PA, USA; cat# 00552-500). Tissues were sectioned at 70 nm, stained with uranyl acetate and lead citrate (Millipore/Sigma, St. Louis, MO; cat# 15326) and imaged using a JEOL JEM1400 PLUS transmission electron microscope (JEOL USA, Inc., Boston, MA, USA) equipped with a Gatan One View digital camera (Gatan, Pleasanton, CA, USA).

### 2.6. Measurements/Quantification of Myelin and Axonal Endpoints

Transversely sectioned tissues were used for quantifying percent of unmyelinated axons, g ratios and myelin and axonal integrity. Longitudinally sectioned tissues were used to assess node length and presence of transverse bands. For the percent of unmyelinated axons, the total number of axons greater than 0.3 µm in diameter [50] was determined from a minimum of 10 electron micrographs collected at 5000×. The number of axons that lacked at least a single complete ensheathment of an oligodendrocyte process, defined as unmyelinated, was determined. To calculate the percentage of unmyelinated axons, we divided the number of unmyelinated axons by the total number of axons per mouse. For g ratio analysis, the same electron micrographs were used. Avoiding areas of fixation artifact, myelin pathologies and inner and outer myelin tongues and avoiding axons not in cross section, as determined by the orientation of the neuronal cytoskeleton, the shortest and longest diameter for each myelinated axon was measured and the widths of the thinnest and thickest regions of the myelin sheath were recorded. Total fiber diameter was calculated by adding the average axon diameter to the two myelin thickness measurements. To calculate the g ratio, the axon diameter was divided by total fiber diameter. A minimum of 100 myelinated axons per mouse was used for g ratio calculation. The average g ratio per mouse constituted an “*n*” for statistical comparison and used for analysis. In addition, all g ratios were plotted, individually per treatment group and per time point, to compare the relative subpopulations of myelinated axons with standard (defined as g ratios between 0.70 and 0.85) or very thin (>0.85) [51] myelin sheaths. Using all 10 electron micrographs collected in the transverse orientation resulting in the analysis of at least 600 axons per mouse, the extent of myelin and axon pathology was quantified as previously described [52]. Briefly, the percent of myelinated axons that presented with specific myelin and axonal pathologies (vacuolar degeneration; redundant myelin profiles, non-compacted myelin sheaths and axonal degeneration) was calculated for each mouse and the total percent of the combined pathologies was determined per mouse.

Transverse band integrity was qualitatively assessed using longitudinally sectioned samples. A minimum of 10 nodes of Ranvier with accompanying paranodes per mouse was used for this analysis. Only the middle two thirds of each paranode were used for transverse band analysis since transverse bands are frequently, and normally, not formed between the nodal-most and juxtaparanodal-most lateral loops and the axolemma [53]. Nodal length was determined by measuring the distance between the most nodally positioned lateral loops of adjacent myelin segments as previously described [30]. For all ultrastructural analyses, 4–7 mice per treatment and time point were used, and all analyses were limited to the ventral columns of the cervical spinal cord. Quantitative analysis for g ratios and node length was conducted using Image J version 1.44.

### 2.7. Immunohistochemistry

Mice 3, 6 and 11 months PI were prepared and imaged as previously described [54,55]. Briefly, mice were perfused as described above with the exception that the perfusion fixative did not contain glutaraldehyde. Cervical spinal cords were cryopreserved in 30% sucrose in 1× phosphate buffered saline (PBS), frozen in Tissue Tek Optimal Cutting Temperature media (Sakura, Torrance, CA, USA) [54], sectioned at 40 µm and triple immunolabeled with antibodies directed against voltage-gated sodium channel type 1.6 (Nav1.6; 1:250, mouse monoclonal IgG1, Antibodies Inc., Davis CA, USA; cat# 75-026), Caspr1 (1:500, rabbit polyclonal, Abcam cat# ab34151) and voltage-gated potassium channel type 1.1 (Kv1.1; 1:750; mouse monoclonal IgG2b, Antibodies Inc., cat# 75-105). Sections were imaged using a Zeiss LSM 880 laser scanning confocal microscope (Oberkochen, Germany). Images were collected using a 63× oil-immersion objective (N.A. of 0.55) employing Nyquist sampling, with a digital zoom of 2, pin hole of 1 Airy unit, optical slice thickness of 0.21 µm and line scan average of 4. X, Y and Z dimensions were 67 µm × 67 µm × 3 µm, respectively. In total, 6–9 mice per genotype per time point were used. With a blinded approach, Image J analysis software version 1.44 was used to assess nodal/paranodal/juxtaparanodal abnormalities by marking overlapping fluorescent labels from maximum intensity projection images.

### 2.8. In Situ Detergent Extraction

In situ protein extraction was performed as previously described with some modification [27]. Briefly, cervical spinal cords from 3, 6 and 11 months PI mice were harvested, diced into small pieces and incubated in extraction buffer consisting of 20 mM Tris-HCl (pH 8.0), 10 mM EDTA, 150 mM NaCl and a protease inhibitor cocktail (Millipore/Sigma, St. Louis, MO, USA, cat# 539136) with or without Triton X-100 (1%; non-ionic) (Millipore/Sigma, St. Louis, MO, USA, cat# X100-5ML) at 4 °C for two hours with constant gentle shaking. After two hours, tissue was removed from the extraction buffer and rinsed in 1XPBS. Optimal incubation time for detergent penetration was determined to be two hours as indicated by Pomicter et al. (2013). Tissue was homogenized in 200 µL of 1% sodium dodecyl sulfate (SDS) (Millipore/Sigma, St. Louis, MO, cat# L3771) in PBS with 5 µL of protease inhibitor cocktail (Millipore/Sigma, St. Louis MO, cat# 539136) for one minute. Homogenates were aliquoted and stored at −80 °C.

### 2.9. Western Blot Analysis

For Western blot analysis, tissue aliquots were thawed on ice and protein concentrations were determined with the Micro BCA Protein Assay kit (Thermo Scientific, Rockford, IL, USA, cat# 23235). Furthermore, 2× Laemmeli Sample Buffer (Bio-Rad, Hercules, CA, USA; cat#1610737) containing 5% β-mercaptoethanol (Millipore/Sigma, St. Louis, MO, USA, cat# M6250) was added to aliquots at a 1:1 ratio. Samples were heated at 70 °C for five minutes. In total, 30 µg of protein was used per sample, except for MBP, where 10 µg of protein were used. Samples were run on 4–20% tris/glycine gels (Bio-Rad, cat# 4561094, Hercules, CA, USA) for 30 min at 70 V followed by an additional 25 min at 150 V. For separation of Neurofascin 155 high and low, proteins were run on 10% gels (Bio-Rad, Hercules, CA, USA, cat# 4561034) for 30 min at 70 V and then 120 min at 150 V, as previously described [26,27]. The protein ladder used was Chameleon Duo pre-stained protein ladder (LICOR, Lincoln, NE, USA, cat#928-60000). Samples were transferred to Immobilon-P transfer membrane (Millipore, St. Louis, MO, USA, cat#IPVH00010) for one hour at 100 V on ice. Membranes were rinsed briefly in 1X PBS with 0.1% Tween-20 (Millipore/Sigma, St. Louis, MO, USA, cat#1379) (PBS-T) and stained for total protein with Revert 700 Total Protein Stain (Lincoln, NE, USA, cat#926-11011). Total protein was visualized using an Odyssey Clx LICOR system (LI-COR, Lincoln, NE, USA, cat#1928) with Image Studio software version 5.2 (LICOR Lincoln, NE, USA) for normalization. Total protein stain was washed off with REVERT Reversal Solution (LICOR, Lincoln, NE, USA, cat#629-11013) and blocked for one hour in 2.5% dry milk in PBS-T. The blot was briefly washed with PBS-T and incubated overnight at 4 °C in Intercept Blocking Buffer (LICOR Lincoln, NE, USA, cat#927-70001) with the appropriate antibody (listed below). The following day, the primary antibody was discarded and the blot was rinsed in PBS-T and incubated with secondary antibody for one hour at room temperature. After one-hour incubation in the appropriate secondary antibody, the blot was rinsed in PBS-T and Western blot was visualized with Odyssey Clx LICOR system. Blots were analyzed using Image Studio software version 5.2.

### 2.10. Antibodies Used for Western Blot Analysis

The primary antibodies used for Western blot analysis are as follows: pan anti-Neurofascin (1:500) (R&D Systems, Minneapolis, MN, USA, cat#AF3235); anti-Myelin-Associated Glycoprotein (MAG) (1:500) (Thermo Scientific, Rockford, IL, USA, cat#34-6200); anti-Myelin Basic Protein (MBP) (1:100) (Millipore/Sigma; St. Louis, MO, USA, cat#MAB386); anti-Cyclin Nucleotide Phosphodiesterase (CNP) (1:500) (Biolegend cat#836404); anti-Myelin Oligodendrocyte Glycoprotein (MOG) (1:500) (Millipore, St. Louis, MO, USA, cat#MAB5680); anti-Glyceraldehyde 3-phosphate dehydrogenase (GAPDH) (1:10,000) (Millipore, St. Louis, MO, USA, cat#MAB374); anti-contactin-associated protein 1 (Caspr1) (1:500) (Abcam Cambridge, UK cat# ab34151). Secondaries from LICOR were diluted at 1:10,000 and are as follows: IRDye 680 anti-rabbit (cat# 926-68073); IRDye 800 anti-rat (cat# 925-32219); IRDye 680 anti-mouse (cat# 926-68070); IRDye 800 anti-chicken (cat#926-32218).

### 2.11. Statistical Analysis

All statistical analyses were performed and graphs generated utilizing GraphPad Prism version 9.4.1 for Windows (GraphPad Software, San Diego, CA, USA). For comparison of sulfatide levels, rotarod performance, percent myelinated/unmyelinated fibers, g-ratios, myelin integrity, axon domains and proteins extraction, two-way ANOVAs were used. For statistical testing of the nodal length (performed by Dr. Leroy Thacker of the VCU Biostatistics Department), the fit of the two models (single variance/covariance matrix or group specific variance/covariance matrices) was compared using a likelihood ratio test (LRT). For all analyses, an α = 0.05 was used for statistical significance and SAS v9.4 was used.

## 3. Results

### 3.1. Confirmation of Gene Ablation and Sulfatide Depletion

The CST floxed mouse was generated with loxP sites flanking the coding regions of the CST gene (Figure 2B). Figure 2C confirms successful CST gene ablation in the spinal cord, which is the region of the CNS used in the present study. Amplification of the ablated gene results in a PCR product of 432 bp and amplification of the unablated gene results in a PCR product of 246 bp. As shown in Figure 2D, mass spectrometric analyses reveal no significant sulfatide reduction by 3 months PI; however, there is ~50% reduction by 6 months PI and ~70% reduction by 11 months PI. This significant reduction of sulfatide confirms that the CST floxed mouse is a viable model for analyzing adult-onset depletion of sulfatide in the spinal cord.

### 3.2. Deficits in Motor Function

**Figure 3:**
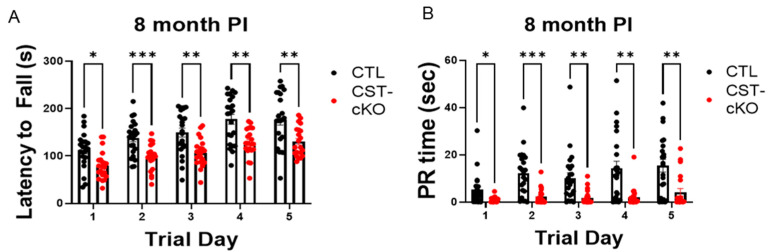
CST-cKO mice demonstrate motor dysfunction through decreased latency to fall and decreased PR time. CST-cKO mice have a significantly shorter latency to fall (**A**) and passive resting (PR) (**B**) time compared to CTL across all trial days (* *p* < 0.05, ** *p* < 0.01, *** *p* < 0.001).

To quantify the extent of functional motor pathology, we used the rotarod paradigm to assess motor function. Using 8 month PI mice with an *n* = 21–23 per genotype, we observed a significant decrease in latency to fall in the CST-cKO mice on all trial days (Day 1 CTL = 112.6 ± 8.2 s (s) versus CST-cKO = 80 ±6.4 s, *p* = 0.0161; Day 2 CTL = 137.7 ± 7.5 s versus CST-cKO = 97.8 ± 6.0 s, *p* = 0.0009; Day 3 CTL = 149.3 ± 9.5 s versus CST-cKO = 107.2 ± 6.4 s, *p* = 0.0038; Day 4 CTL = 177.5 ± 10.3 s versus CST-cKO = 128.3 ± 6.6 s, *p* = 0.0014; Day 5 CTL = 177.2 ± 11.7 s versus CST-cKO = 130.1 ± 6.8 s, *p* = 0.0068; Figure 3). In addition, we observed a significant decrease in passive rotation (PR) time in the CST-cKO mice, which is the amount of time the mouse did not move on the rotarod (Day 1 CTL = 5.17 ± 1.45 s (s) versus CST-cKO = 0.82 ± 0.27 s, *p* = 0.0356; Day 2 CTL = 12.25 ± 2.02 s versus CST-cKO = 2.35 ± 0.70 s, *p* = 0.0004; Day 3 CTL = 10.185 ± 2.3 s versus CST-cKO = 1.86 ± 0.66 s, *p* = 0.0077; Day 4 CTL = 14.33 ± 3.00 s versus CST-cKO = 2.08 ± 0.91 s, *p* = 0.0029; Day 5 CTL = 15.45 ± 2.69 s versus CST-cKO = 4.17± 1.53 s, *p* = 0.0045; Figure 3).

### 3.3. Myelin Thinning and Axon Ultrastructural Pathology

**Figure 4:**
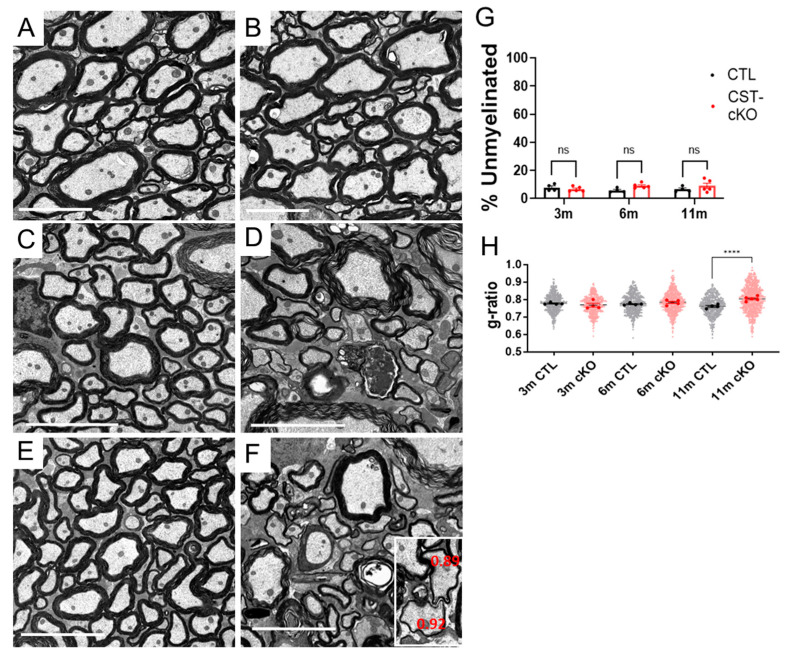
Sulfatide depletion resulted in no change in the percent of unmyelinated axons and myelin thickness was only reduced at 11 months PI. (**A**) Cross section representative images from the ventral columns of the spinal cord from control (CTL; **A**,**C**,**E**) and CST-cKO (**B**,**D**,**F**) mice following 3 (**A**,**B**), 6 (**C**,**D**) and 11 (**E**,**F**) months post injection (PI). Mice 3, 6 and 11-months PI presented abundant myelinated axons with no difference in the percent of myelinated versus unmyelinated fibers (**G**). However, we observed an increase in g-ratios at 11 m PI, indicating demyelination/remyelination (**H**). Note the presence of axonal pathology in the 6-m- and 11-m-PI CST-cKO mice as well as evidence of demyelination/remyelination at 11 m PI (**F** inset; g-ratios greater than 0.85 indicate remyelination). Data are presented as mean ± standard error. Scale bar = 5 µm. **** *p* < 0.0001.

To quantify the consequence of adult-onset sulfatide depletion on myelin structure, we initially determined the percent of unmyelinated axons in the ventral column of the cervical spinal cord of CST-cKO and CTL mice 3, 6 and 11 months PI (*n* = 4–6 and a minimum of 650 axons quantified per animal). No difference in the percent of unmyelinated axons was observed at any of the survival time points (3 m CTL = 7.9 ± 1.2 versus CST-cKO = 6.6 ± 0.8, *p* = 0.903; 6 m CTL = 5.8 ± 0.7 versus CST-cKO = 9.0 ± 0.8, *p* = 0.316; 11 m CTL = 6.5 ± 1.0 versus CST-cKO = 10.3 ± 2.1, *p* = 0.16; Figure 4). We have previously reported that ~7% of the axons in the ventral column of the cervical spinal cord of c57black6/J wild type mice are unmyelinated [30]. Therefore, these counts are consistent with previous analysis [30].

**Figure 5:**
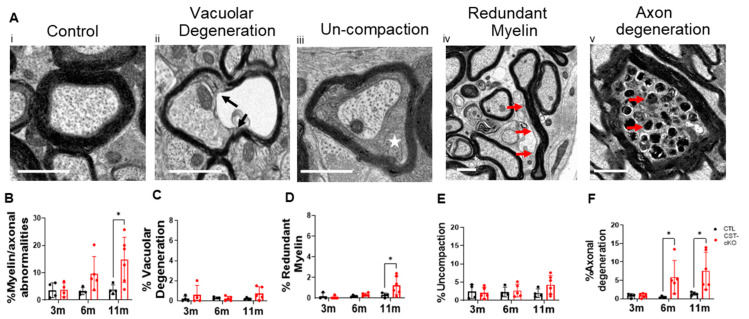
Quantification of myelin and axonal pathologies in the dorsal region of the cervical spinal in CST-cKO mice. (**A**) Representative image of a healthy axon with an intact, compacted myelin sheath and an appropriate array of neurofilament and microtubules (**Ai**). Representative images of various myelin and axonal abnormalities observed in the spinal cord of the CTL and CST-cKO mice: (from left to right): axon losing attachment to the myelin sheath (**Aii**; vacuolar degeneration; black arrows); presence of cytoplasm between compacted wraps of myelin (**Aiii**; un-compaction; white star); compact myelin out folding (**Aiv**; redundant myelin; red arrows) and organelle fluid filled swellings and cytoskeletal degeneration within the axon (**Av**; axon degeneration; red arrow). (**B**) Quantitative analysis combining all pathologies revealed a significant increase in myelin/axonal pathologies at 11 m PI but not at 3- and 6- months PI. Separating out the individual pathologies revealed no significant difference in myelin ultrastructure between genotypes at any of the time points analyzed (**C**,**D**) or only at the latest time point (**E**); however, (**F**) axonal degeneration was significantly increased in the 6-m- and 11-m-PI CST-cKO mice. * *p* < 0.05.

**Figure 6:**
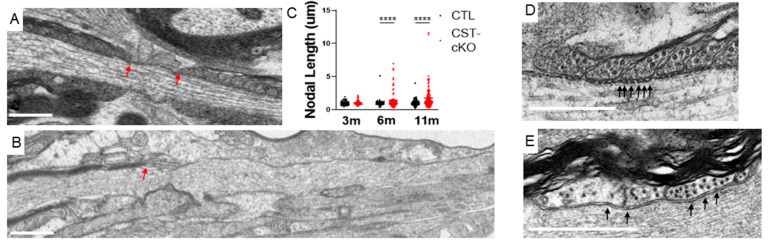
Increase in nodal length variability and loss of transverse bands following adult-onset sulfatide depletion. (**A**) Normal ultrastructure of a node of Ranvier (indicated by red arrows) including nodal length (~1 micron) and flanking paranodal regions with lateral loops closely associated to the axolemma. (**B**) CST-cKO mice at 6 and 11 months PI revealed nodes of Ranvier with varying lengths including abnormally long nodal gaps. Heminodes, defined by a single paranode (indicated by a red arrow) with no adjacent paranode in the plane of a section for at least 3 microns, were also observed. (**C**) Overall, node quantitation revealed no significant change in average nodal length between genotypes at any of the time points analyzed; however, a significant difference was observed in length variability in the 6 and 11 months post tamoxifen injected CST-cKO mice with nodes of Ranvier presenting both shorter and longer than 1 micron. (**D**) CTL mice revealed equally spaced electron densities (arrows), at the interface between the lateral loops of the myelin sheath and the axolemma. (**E**) In contrast, transverse bands were missing and structurally disrupted (note electron densities without clearly defined edges, arrows) in the periaxonal space of the CST-cKO mouse. For analysis, the fit of the two models (single variance/covariance matrix or group specific variance/covariance matrices) was compared using a likelihood ratio test. (**A**,**B**) Scale bars = 1 µm. **** *p* < 0.0001. (**D**,**E)** Scale bars = 0.5 µm.

Myelinated axons from the spinal cords of the CST-cKO mice presented with no difference in myelin thickness, as quantified by g ratios at 3 months PI (*n* = 4 and 4 for CTL and CST-cKO, respectively, a minimum of 108 axons per animal) (CTL = 0.779 ± 0.003 vs. CST-cKO = 0.770 ± 0.011; *p* = 0.67; Figure 4A,B) and 6 months PI (*n* = 4 and 5 for CTL and CST-cKO, respectively, a minimum of 110 axons per animal) (CTL = 0.774 ± 0.003 vs. CST-cKO = 0.790 ± 0.005; *p* = 0.22; Figure 4C,D). However, by 11 months PI, a modest but significant increase was observed in g ratios (thinner myelin) (*n* = 4 and 5 for CTL and CST-cKO, respectively, a minimum of 103 axons per animal) (CTL = 0.763 ± 0.006 vs. CST-cKO = 0.804 ± 0.006; *p* = 0.002; Figure 4E,F). Although most myelinated axons revealed myelin thickness in the normal range, based on axon caliber (g ratios between 0.70 and 0.85), a subset of the axons in the 11 months, PI CST-cKO mice, presented with abnormally thin myelin sheaths (g ratios > 0.85) [56]. Axons with very thin myelin sheaths are presented in Figure 4F (inset) and the prevalence of axons with extremely thin myelin sheaths is indicated by the scatter plot in Figure 4H. Next, to analyze the integrity of myelinated axons, we quantified frequency of myelin non-compaction, vacuolar degeneration, redundant myelin and axonal degeneration (Figure 5A). At 3 months PI, there was no difference in integrity of the myelinated axons between the sulfatide deficient and control mice (CTL = 3.6% ± 1.4%; versus CST-cKO = 3.8% ±1.3%; *p* = 0.99). By 6 months PI, there was an apparent progressive increase in compromised integrity (CTL = 3.2% ± 0.6%; versus CST-cKO = 9.7% ± 2.8%; *p* = 0.21) that reached significance by 11 months PI (CTL = 3.9% ± 0.9%; versus CST-cKO = 14.7% ± 3.4%; *n* = 6; *p* = 0.01) (Figure 5B). Interestingly, when we graphed these individual pathologies, we did not see a significant difference in myelin non-compaction nor myelin vacuolar degeneration, but we did see a significant increase in redundant myelin (Figure 5E) at 11 m PI as well as axonal degeneration both at 6 and 11 months PI (Figure 5F). These data indicate that axonal pathology occurs independent of myelin loss with possible demyelination/remyelination occurring at 11 m PI. Although it is not possible to definitively state that demyelination/remyelination has occurred, the presence of very thin myelin sheaths (g ratios > 0.85) provides strong supportive evidence of remyelination (Figure 4F inset) [56]. Demyelination followed by remyelination also provides an explanation for the overall increase in g ratios at 11 months PI.

### 3.4. Compromised Nodal Structure

Considering that the constitutive CST KO mice presented with altered node/paranode organization, [30] we next analyzed these domains in the CST-cKO mice. In contrast to our findings in the constitutive CST KO mice, we did not observe everted or disorganized lateral loops in the paranode at any of the time points analyzed. Although not observed in the mice 3 months PI, qualitative assessment revealed frequent disruption of transverse bands in the periaxonal space of 6 and 11 months PI mice (Figure 6B). Additionally, we analyzed the length of the nodal gap (*n* = 4–6 mice per genotype, minimum of 10 nodes per animal). At 3 months PI, the nodal length was similar between the treatment groups (CTL = 1.20 µm ± 0.33 µm; nodes versus CST-cKO = 1.08 µm ± 0.34 µm; *p* = 0.74). By 6 and 11 months PI, we observed an increase in variability, with the CST-cKO having both longer and shorter nodes (Figure 6C). The variance test statistically confirmed that 6 and 11 months PI mice had a significant difference in variability (*p* < 0.001) (Figure 6C).

### 3.5. Compromised Molecular Organization of the Nodal Regions

**Figure 7:**
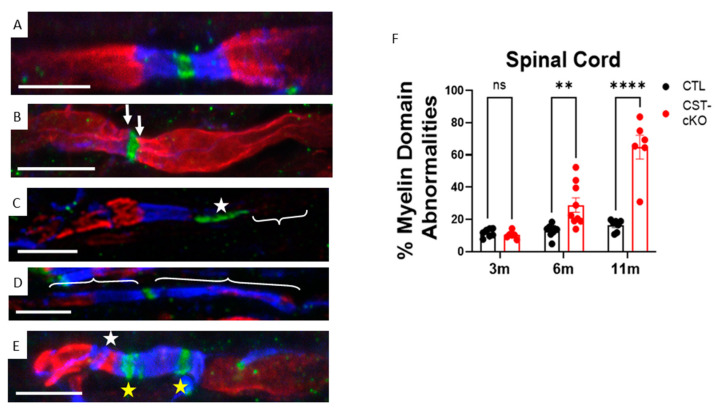
Mislocalization of axonal domain proteins following adult-onset sulfatide depletion. (**A**) An axon from a CTL mouse demonstrates proper ion channel and axonal domain protein organization in the spinal cord with Nav1.6 (green) localized to the node of Ranvier, Caspr1 (blue) localized to the paranode and Kv1.1 (red) localized to the juxtaparanode. Common, but not exclusive, pathologies observed included (**B**) Kv1.1 invading the paranodal region (white arrows); (**C**) elongated sodium channel domain (white star) and heminode (white bracket) (note: z-stack collected encompassed entirety of fluorescence and absent paranode was not the result of exclusion of z-stack); (**D**) elongated Caspr1 localization (white brackets); (**E**) combination of several pathologies including binodal sodium channels clusters (yellow stars) and intermixing of Kv1.1 channels and Caspr1 (white star). (**F**) Quantification of protein organization revealed preservation of protein domains at 3 months PI but a significant loss of axonal domain organization at 6 and 11 months PI. Data are presented as mean ± standard error; ** *p* < 0.01 **** *p* < 0.0001. Scale bars = 5 µm.

Together, demyelination followed by remyelination, as indicated by g ratios greater than 0.85, disruption of transverse bands, axonal pathology and variable nodal gap length are consistent with compromised axonal domain organization. To directly quantify domain organization, we employed triple immunohistochemical labeling. As shown in Figure 7, Nav1.6, Caspr1 and Kv1.1 are restricted to their appropriate axonal domains, which are the node of Ranvier, the paranode and the juxtaparanode, respectively, in the CTL mice. While there was no significant difference at the 3-month time point (CTL = 11.6% ± 1.0%; versus CST-cKO = 10.3% ± 0.9%; *p* = 0.99), which is consistent with sulfatide’s turnover rate, a significant increase in domain disruption was observed at 6 m PI (CTL = 12.9% ± 1.2%; versus CST-cKO = 28.9% ± 4.5%; *p* = 0.003) and 11 m PI (CTL = 16.3% ± 1.4%; versus CST-cKO = 64.8% ± 7.4%; *p* < 0.0001; Figure 7). We observed a significant and progressive deterioration of the protein domains highlighted by an invasion of Kv1.1 into the paranode (Figure 7B); Nav1.6 elongation without evidence of either a downstream paranode or juxtaparanode (Figure 7C), which is suggestive of heminode formation, Caspr1 length elongation (Figure 7D); bi-nodal labeling of Nav1.6 and intermixing of Kv1.1 channels and Caspr1 (Figure 7E). This loss of protein domain organization is consistent with previous findings from the constitutive CST KO mice [26,27,57].

### 3.6. Disruption of Membrane Stability

**Figure 8:**
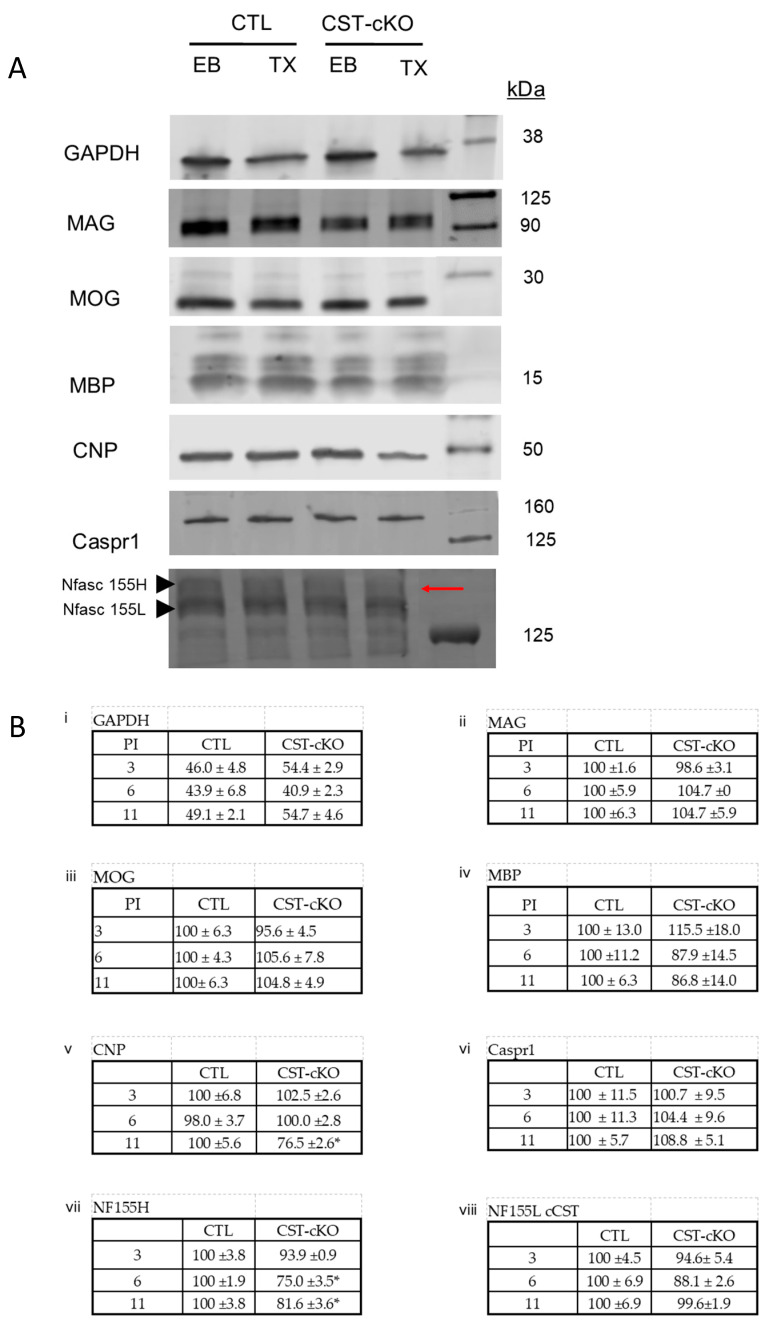
(**A**) Nfasc155H and CNP but not MAG, MOG, MBP and Caspr1 of Nfasc155 L exhibit an increased susceptibility to detergent extraction in the absence of sulfatide. Cervical spinal cords from CTL and CST-cKO mice, incubated in extraction buffer without detergent (EB) or in buffer containing detergent Triton X-100 (TX). GAPDH (38 kDa) was extracted independent of sulfatide at a consistent ratio across time points to confirm consistency of technique. Nfasc155H (155 kDa) and CNP (48 kDa) were extracted at a greater rate in the CST-cKO mice (red arrow is pointing to Nfasc155 H), while MAG (100 kDa), MOG (28 kDa) and MBP isoforms (21.5, 18.5, 17.0, and 14.0 kDa), Nfasc155 L (155 kDa) and Caspr1 (156 kDa) remain unchanged regardless of treatment or genotype. (**B**) Values (mean ± SEM) indicate percent of protein remaining after Triton X-100 incubation compared to spinal cords incubated without detergent. GAPDH extracts consistently across all time points and genotypes. MAG, MOG, MBP, Caspr1 and Nfasc155L are not dependent on sulfatide for stability in the membrane. Nfasc155H and CNP are significantly more extracted in CST-cKO mice. * indicates *p* < 0.05. Note that all isoforms of MBP that were detected (21.5, 18.5, 17.0 and 14.0 kDa MBP isoforms) were quantified. See appendix figure (Figure A1) for total protein stains.

Based on these observations, and consistent with our previous work [27], we hypothesized that the reduction of sulfatide in the myelin membrane resulted in less-stable anchoring of specific proteins within the sheath. To test this hypothesis, we used a published extraction method of exposing tissue to 1% Triton-X 100 [27,32,58]. To assess the effectiveness of detergent penetration, we used GAPDH, a cytoplasmic protein, to compare tissue exposed to no triton versus tissue exposed to 1% Triton-X 100 and found that ~50% of GAPDH was consistently extracted independent of time point or genotype (Figure 8). Upon confirming the validity of this approach, we applied this method to our CTL and CST-cKO tissues and found no difference in the membrane stability of MBP, MOG, MAG, Nfasc155 low or Caspr1 regardless of genotype or time PI (Figure 8). In contrast, Nfasc 155 high was significantly more susceptible to extraction in the CST-cKO mice at 6 and 11 months PI (72.9% protein remaining, *p* = 0.0002 and 82.6% protein remaining, *p* = 0.0024, respectively) (Figure 8B), while CNP was more susceptible to extraction at 11 months PI (71.5% protein remaining, *p* = 0.0014) (Figure 8).

## 4. Discussion

Confirming our previous report [45], we show that the CST floxed mouse provides a viable model for inducing significant and consistent adult-onset depletion of sulfatide in the CNS. Following tamoxifen injection, crerecombinase is translocated into the nucleus resulting in excision of the floxed region of the gene. Since whole spinal cord was used as the starting material, both ablated and unablated products are expected, since non-oligodendrocytes present non-ablated genes. The presence of the unablated product could also result from less than 100% efficient oligodendrocyte gene ablation. The use of whole spinal cord precludes quantifying oligodendrocyte ablation efficiency; however, the true indicator of success of the approach is the analysis of sulfatide levels.

After observing a motor defect in the adult-onset sulfatide deficient mice, we analyzed myelin and axonal protein domains in the spinal cord ventral columns, which houses the axons of the ventral horn motor neurons. Furthermore, we employed a previously published biochemical extraction approach [27,58,59] to begin to elucidate the mechanistic consequence of sulfatide loss on myelin sheath integrity. In our studies, we observe both ultrastructural and molecular differences between the constitutive CST KO mice and the adult-onset sulfatide deficient model. The constitutive CST KO mouse displays more profound motor deficits as evidenced by the development of whole body tremor by 6 weeks of age [43]. Accompanying the severe motor dysfunction is the near-complete loss of transverse bands and overt myelin pathologies. In contrast, the CST-cKO mouse presents relative sparing of the myelin sheath (Figure 4 and Figure 5). It remains to be determined why the two models present with differences in functional deficits and myelin sparing. Possible explanations include: 1. total (CST KO) versus partial sulfatide (CST-cKO) depletion, 2. impaired myelin development (CST KO) versus normal myelin development (CST-cKO) and 3. age-dependent differential functions of the lipid.

### 4.1. What Proteins Rely on Sulfatide for Myelin Sheath Tethering?

We demonstrate that CST-cKO mice have a shorter latency to fall, which is indicative of the compromised function of the motor neurons. Similarly, motor dysfunction was observed in the constitutive CST KO, although this motor dysfunction was much more pronounced, having a visible tremor at 6 weeks of age [43]. Our initial ultrastructural studies demonstrated that adult-onset loss of sulfatide leads to axonal pathology with relative sparing of the myelin sheath. This is in contrast to the constitutive CST KO mice, which showed an increase in g-ratios as early as 15 days of age [30] as well as an increase in myelin pathologies, such as vacuolar degeneration, redundant myelin and a progressive increase in uncompacted myelin, which were first observed at 1 month of age. In order to understand this disparity of sulfatide dependence on myelin sheath integrity during development versus adulthood, we utilized the triton extraction approach to perturb the membrane. The Triton-X100 detergent extraction method selectively removes proteins and lipids from the membrane that are not securely tethered in place [32,58]. This approach provides a biochemically based strategy to identify membrane proteins that are differentially bound within the membrane.

The extraction method showed both genotypic and age-related differences between the constitutive CST KO and the conditional CST-KO mouse. The constitutive CST KO mouse, at 15 days of age, showed an effect of triton extraction independent of genotype for MOG with the protein being equally susceptible to extraction in both the WT and constitutive CST KO mice [27]. However, MOG was not susceptible to extraction in either the CTL or the CST-cKO mice at any of the PI times analyzed. MOG is located in the extracellular leaflet [60] of the myelin sheath, potentially facilitating ease of extraction. Additionally, MOG has been reported to be incorporated into myelin lipid rafts (reviewed by Taylor et al. (2002), which could potentially be disrupted in the absence (or at least depletion) of sulfatide [32]. However, classes of rafts present with differential lipid and protein compositions and as reported by Arvanitis et al. (2002), a greater proportion of MOG is recruited into lipid fractions that are sulfatide-poor, suggesting a reduced dependency on sulfatide for MOG’s tethering in the membrane [38,61].

Unlike MOG, MAG was susceptible to detergent extraction in the adolescent constitutive CST KO mice but extraction required sulfatide depletion [27]. In contrast, MAG was not readily extracted from either the CTL or the adult-onset sulfatide depleted mice. This demonstrates that while MAG is sulfatide dependent for proper stabilization into the myelin sheath during development, MAG employs sulfatide-independent means to stabilize in the adult myelin sheath. Similar to MOG, MAG is also present in greater abundance in the sulfatide-poor lipid fractions (Arvanitis et al., 2002), which again supports our observation that membrane tethering of MAG does not require sulfatide. It is important to note that the starting material used by Arvanitis et al. was adult CNS, which is consistent with the CST-cKO mouse starting material and leaves open the possibility that MAG has an age-specific dependency on sulfatide for membrane tethering. Other possible explanations for the limited extractability of MAG include its presence in the inner most wrap of the myelin sheath reducing its availability to the detergent and its potential binding to axonal gangliosides such as GD1a and GT1b [41,62,63]. Perhaps in the mature myelin sheath, MAG’s binding to axonal ligands provides additional and age-specific tethering that stabilizes the protein in the myelin sheath.

In contrast to the constitutive CST KO mouse studies, which revealed no CNP extraction [27]. CNP was readily extracted at the 11-month-PI time point from the CST-cKO mice, consistent with the employment of differential stabilization mechanisms in the developing and adult myelin sheath. CNP is commonly found in Triton-X 100 insoluble lipid rafts [64] and our studies confirmed CNP is resistant to extraction at the 3- and 6-month time points in both CTL and CST-cKO. However, at the 11-month time point, we see a significant extraction of CNP in the CST-cKO mice. CNP is localized to the regions of non-compact myelin, particularly the leading edge, where sulfatide is enriched [65,66,67,68]. CNP has post-translational modifications including iso-prenylation and phosphorylation, [69,70], which may aid in its incorporation into and stability within lipid rafts, and also has associations with the underlying cytoskeleton [71]. CNP is an early oligodendrocyte marker and is expressed in pre-myelinating oligodendrocytes [72]. During process extension, CNP associates with microfilaments, but in later stages, once membranes sheets have formed, CNP co-localization with microfilaments disappears [73]. Perhaps once process extension is complete and the microfilaments depolymerize, CNP remains in the non-compact myelin via sulfatide associations.

CNP’s instability in the myelin sheath due to sulfatide loss may contribute to the observed pathologies. CNP is a part of the myelinic channels, such as the leading edge, which are known to support axons [74]. Perhaps disruption of the axo-glial complex at the level of CNP alters feedback mechanisms for the oligodendrocyte [75], resulting in axolemmal disorganization. Furthermore, CNP-null mice exhibit structurally intact myelin with subsequent axonal pathology [76,77]. Thus, while CNP is still being expressed, perhaps its instability is sufficient to contribute to the axonal pathology presented by the CST-cKO animals.

Similar to the constitutive CST KO mice, our data show that Nfasc 155 high (H) is more susceptible to extraction from the CST-cKO mice than from the CTL mice. Our lab discovered that Nfasc 155 exists in two forms that we identified as Nfasc155H and Nfasc 155 low (L) [26]. While it is unknown if Nfasc 155 H and L have different functions or distributions, Nfasc 155 H may be more readily incorporated into lipid rafts. Lipid rafts are enriched in cholesterol and glycosphingolipids that contain long acyl carbon chains [78,79]. Neurofascin proteins contain post-translational modifications, such as palmitoylation, which facilitates stability of Nfasc in rafts since cleavage of palmitate residues from neurofascin facilitated triton-mediated Nfasc155 extraction, while Caspr1 was unaffected [14]. Therefore, the location and post-translational modifications of Nfasc155H likely forms sulfatide-dependent associations within the membrane. We predict that mislocalization of Nfasc 155H at the level of the paranode is responsible for the disruption of transverse bands. If Nfasc155H is not properly stabilized within the myelin sheath, perhaps it is unable to find its binding partner, therefore leading to disruption of the axo-glial complex. This possibility is supported by the gradual loss of Caspr1 clustering in adult-onset depletion of Nfasc155 [80,81].

### 4.2. Consequence of Myelin Protein Instability

The axo-glial complex is composed of Neurofascin 155 on the myelin side and contactin and Caspr1 on the neuronal side [40]. These proteins sort to their determined location [40,82,83,84,85] and bind to form complexes that appear as electron densities in the paranode. Potentially, as a consequence of protein mislocalization and compromised axo-glial junction formation, myelin sheath tethering is undermined facilitating node length variability in both 6 and 11 months PI mice. In the healthy CNS, the length of the node of Ranvier is highly conserved, about 1 µm [86], and changes in node length significantly alter axon firing [87,88], subsequently leading to motor functional deficits.

Accompanying the change in nodal length is the movement of the ion channels that are specifically clustered in the node and juxtaparanode. Using immunohistochemical labeling, we observed at both 6 and 11 months PI an increase in Caspr1 length (Figure 7D), and elongated and binodal (Figure 7C,E) sodium channel clusters. This is consistent with reduced stability, or presence, of Nfasc 155 within the myelin sheath that has been shown to bind to the contactin-Caspr1 complex and to facilitate compromised ion channel clustering [40,80,81]. Transverse bands, a component of the paranodal junctional complex, provide a mechanism to restrict sodium and potassium channels to their proper domain. Disruption of the transverse bands may lead to the ion channel mislocalization we observed [24]. This is in contrast to our ultrastructure data, which did not show myelin pathology until 11 months PI. Since proper spatial distribution of these ion channels is necessary for proper ion conduction, we predict that this mislocalization contributes to the motor deficits observed in the CST-cKO mice. Based on our findings, it would appear that ion channel mislocalization may be a more sensitive indicator of axonal health and function than either g-ratio measurements or myelin ultrastructural changes, which have been used for assessing myelin and axonal damage in MS patients [89]. Perhaps loss of sulfatide seen in MS may contribute to neurofascin cluster instability, which is a consistent consequence of demyelination [90] and transverse band disruption [91] in the human disease. Moreover, diffuse sodium channel distribution, which is accompanied by altered conduction activity, is sufficient to lead to axonal degeneration [92,93]. Together, our data provide evidence that molecular disorganization of the myelin sheath due to sulfatide loss is sufficient to lead to axonal pathology, and molecular disorganization is the first step before overt myelin pathology and motor dysfunction occur. Therefore, in the case of MS in the NAWM where loss of sulfatide has been reported, lipid dyshomeostasis may be occurring prior to clinical diagnosis, thus defining the prodromic disease period of MS. While further experimental evidence is lacking to prove causality, the loss of sulfatide may present a valuable early marker for disease diagnosis.

## 5. Conclusions

In conclusion, our studies provide evidence of sulfatide-dependent molecular mechanisms that regulate the stability of the axo-glial junction. Loss of sulfatide leads to instability of CNP and Nfasc155 H in the paranode/uncompacted myelin that is accompanied by subsequent axonal pathology with myelin sparing. These studies shed light on early pathogenesis in MS where sulfatide is reduced prior to demyelination consistent with the loss of sulfatide, representing a prodromic period in MS before demyelination.

## Figures and Tables

**Figure A1:**
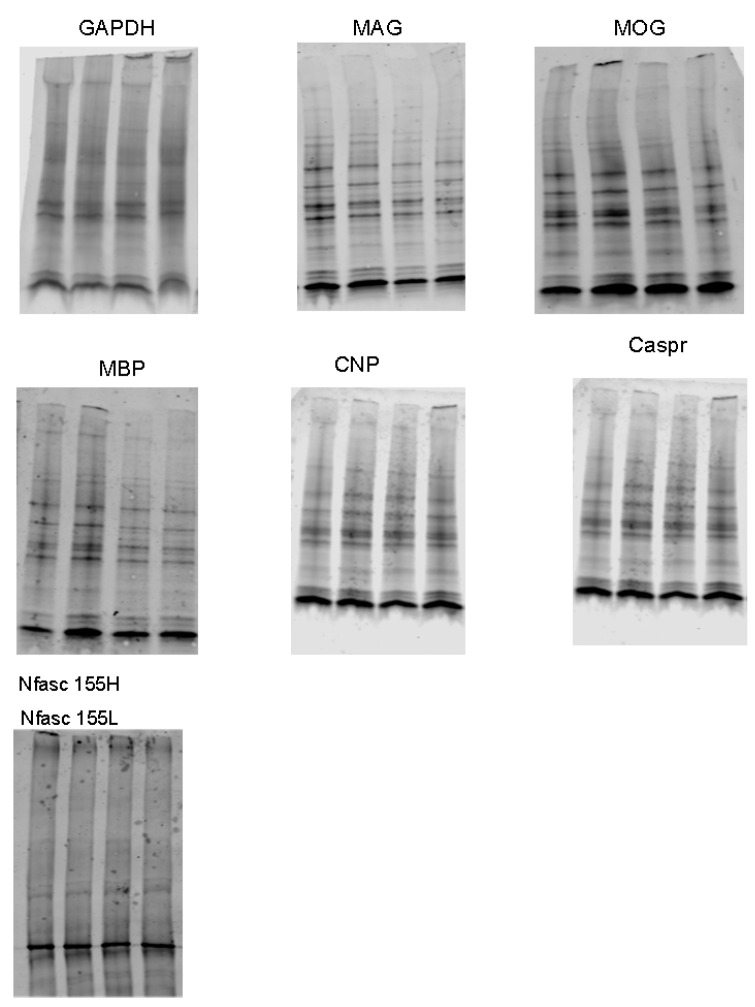
Total protein load for Western blot analysis. Total protein blots, stained with REVERT total protein stain, were used to normalize protein percentages for all proteins analyzed. Note that Nfasc155 total protein is lighter due to different gel conditions, see methods.

## Data Availability

All data and reagents will be made available in accordance to Veterans Affair Material Transfer Agreement Guidelines.

## Abbreviations

Caspr1	Contactin-Associated protein 1
cKO	conditional knock out
CNP	2′:3′-cyclic nucleotide 3′-phosphodiesterase
CNS	Central Nervous System
CST	Ceramide Sulfotransferase
CTL	Control
EM	Electron Microscopy
GAPDH	Glyceraldehyde 3-phosphate dehydrogenase
KO	Knock-Out
Kv1.1	Voltage-Gated Potassium Channel Kv1.1
m	month
MAG	Myelin-Associated Glycoprotein
MBP	Myelin Basic Protein
MOG	Myelin Oligodendrocyte Glycoprotein
MS	Multiple Sclerosis
Nav1.6	Voltage-gated sodium channel Na_v_1.6
NAWM	Normal Appearing White Matter
Nfasc155	Neurofascin 155
Nfasc155H	Neurofascin 155 high
Nfasc155L	Neurofascin 155 L
PI	Post-Injection
PLP	Proteolipid Protein
Sulfatide	3-O-sulfogalactosylceramide
TAG-1	Transient Axonal Glycoproteintype-1
